# Highly skewed distribution of miRNAs and proteins between colorectal cancer cells and their exosomes following Cetuximab treatment: biomolecular, genetic and translational implications

**DOI:** 10.18632/oncoscience.19

**Published:** 2014-03-16

**Authors:** Marco Ragusa, Luisa Statello, Marco Maugeri, Cristina Barbagallo, Roberta Passanisi, Mohamed S. Alhamdani, Giovanni Li Destri, Alessandro Cappellani, Davide Barbagallo, Marina Scalia, Hadi Valadi, Jörg D. Hoheisel, Cinzia Di Pietro, Michele Purrello

**Affiliations:** ^1^ Molecular, Systems and Genome BioMedicine Unit, Department Gian Filippo Ingrassia, University of Catania, Catania, Italy, EU; ^2^ Division of Functional Genome Analysis, Deutsches Krebsforschungszentrum (DKFZ), Heidelberg, Germany, EU; ^3^ Dipartimento di Scienze Chirurgiche, Trapianti d'Organo e Tecnologie Avanzate, Università di Catania, Catania, Italy, EU; ^4^ Dipartimento di Chirurgia, Università di Catania, Catania, Italy, EU; ^5^ University of Gothenburg, Department of Rheumatology and Inflammation Research, Gothenburg, Sweden, EU

**Keywords:** Cetuximab, Colon Cancer, Exosomes, miRNAs, Proteins

## Abstract

Exchange of molecules via exosomes is a means of eukaryotic intercellular communication, especially within tumour microenvironments. However, no data are available on alterations of exosomal molecular cargo by environmental cues (eg, pharmacological treatments). To approach this issue, we compared the abundance of 754 miRNAs and 741 cancer-related proteins in exosomes secreted by Caco-2 (Cetuximab-responsive) and HCT- 116 (Cetuximab-resistant) CRC cells, before and after Cetuximab treatment, with that in their source cells. Cetuximab significantly altered the cargo of Caco-2 exosomes: it increased abundance of miRNAs and proteins activating proliferation and inflammation and reduced miRNAs and proteins related to immune suppression. These alterations did not precisely mirror those in source cells, suggesting a Cetuximab-linked effect. Analogous alterations were detected in HCT-116. Transfection of exosomes from Cetuximab-treated Caco-2 into HCT-116 significantly increased HCT-116 viability; conversely, no viability alteration was detected in Caco-2 transfected with exosomes from Cetuximab-treated HCT-116. Analysis of networks, comprising targets of differentially expressed (DE) exosomal miRNAs and DE exosomal proteins, demonstrates a significant involvement of processes related to proliferation, inflammation, immune response, apoptosis. Our data extend existing knowledge on molecular mechanisms of eukaryotic intercellular communication, especially in oncological processes. Their translation to clinical settings may add new weapons to existing therapeutic repertoires against cancer.

## INTRODUCTION

Exosomes are nanosized vesicles (50-120 nm in diameter), which derive from endosomal compartment invaginations called Multivesicular Bodies (MBVs). MBVs may be released into the extracellular milieu by fusion with the plasma membrane as an alternative to fusion with the lysosomal compartment [1, 2]. Exosomes are synthesized by possibly all cell phenotypes, including tumour cells and platelets [3 - 6]. They also have been found in several body fluids (*eg*, blood, saliva, urine, bronchoalveolar fluid, synovial fluid, amniotic fluid, breast milk, malignant ascite) [7 - 12]. Exosome internalization by recipient cells follows binding of exosomal membrane adhesion molecules to cellular receptors (*eg*, LFA1, TIM1, TIM4) [13, 14]. Exosomes from different cell phenotypes and biological fluids have been shown to contain functional subsets of proteins, mRNAs and miRNAs, which can be transferred to recipient cells: this would suggest a novel and still partially unexplored mechanism of communication and genetic exchange among eukaryotic cells [15 - 19]. Sorting of RNAs and proteins into exosomes is likely a selective process, whose general rules and signals started to be clarified only recently [15, 20, 21]. Notably, the RNA cargo of exosomes may partially diverge from that of source cells: in fact, the same cells if cultured under different conditions (*eg*, serum starvation, oxidative stress) may produce exosomes with different molecular cargos [15, 22]. Accordingly, it would seem logical to hypothesize that nucleic acids and proteins may be selectively sorted into exosomes depending on the specific molecular signals they carry. Exosomes have been shown to be involved in different biological functions, as intercellular communication, modulation of immune functions, transport and propagation of prions and retroviruses [23 - 25]. Tumour-derived exosomes have been found to possess immunosuppressive and protumorigenic properties, since they apparently facilitate tumour growth and metastasis [6, 26]. It has been recently noted that tumour cells possess increased exosomes-shedding properties compared to normal cells [27]. Significantly increased levels of serum exosomes were detected in patients with ovarian cancer, compared to healthy controls [28]. Also, high levels of exosomes in plasma of colorectal cancer (CRC) patients were secreted by poorly differentiated tumours and were associated with decreased overall survival [29]. Despite these scientific advances, the biologic and pathologic significance of exosomes is still not fully understood. Based on these premises, we sought to analyze the alterations of exosomal miRNAs and proteins cargo profiles from CRC cells following Cetuximab treatment. Cetuximab (*Erbitux*) is a monoclonal antibody that acts as competitor of natural ligands of the Epidermal Growth Factor Receptor (EGFR): Epidermal Growth Factor, Amphiregulin, Epiregulin, Transforming Growth Factor α. Accordingly, it is among the most commonly used drugs for treating advanced metastatic CRC [30]. Cetuximab binding to EGFR impairs its activation and leads to a block of downstream KRAS proliferative signalling, thus affecting tumour cells proliferation [30]. However, KRAS mutations in CRC patients may lead to permanently activated EGFR pathway regardless of the EGFR status, so preventing efficacy of Cetuximab-based therapy [31]. In previous work, we have shown that *in vitro* sensitivity of CRC cells to Cetuximab depends on specific miRNA transcriptome profiles [32]. Interestingly, a correlation between exosomes and effectiveness of monoclonal antibody-based therapy has already been found in breast cancer: exosomes secreted by HER2-overexpressing breast carcinoma cells express full-length HER2 molecules on their surface, which bind and sequester Trastuzumab (a therapeutic monoclonal antibody) and lower its therapeutic efficacy [33]. The data reported in this paper demonstrate that Cetuximab significantly alters the miRNAs and proteins cargo of exosomes released by CRC cells. Intriguingly, we also show that transfection of steady-state or Cetuximab-treated HCT-116 (Cetuximab unresponsive) with exosomes from Cetuximab-treated Caco-2 (Cetuximab sensitive) significantly increases HCT-116 viability and alters their Cetuximab responsiveness.

## RESULTS

### Characterization of Exosomes from Caco2 and HCT-116 cells

**Figure 1:**
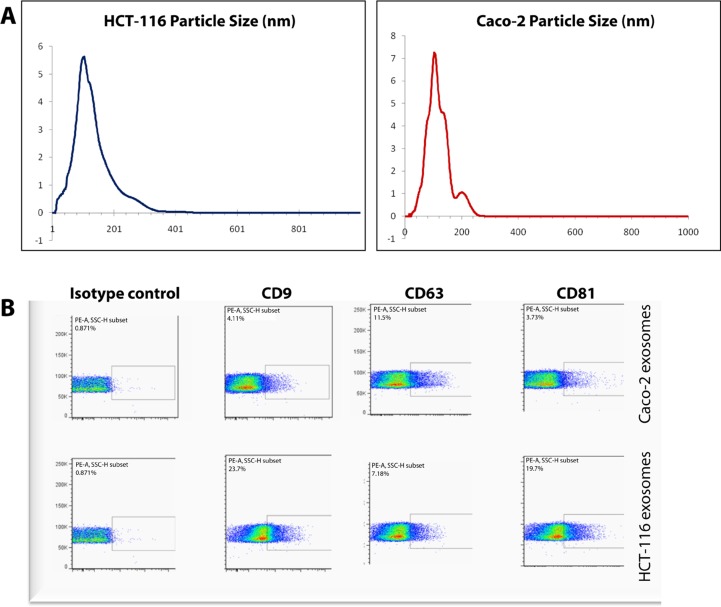
Characterization of Caco-2 and HCT-116 exosomes (A) Average particle sizes in exosome samples were determined by dynamic light scattering. Y-axes: signal intensity (%); X-axes: size of particles (nm). (B) FACS analysis was performed based on exosome markers CD9, CD63 and CD81 on nanoparticles isolated from Caco-2 and HCT-116 cells.

Following exosome isolation, the size of pelleted particles was determined with dynamic light scattering using a Zetasizer Nano. The results showed that the pellet consisted of particles with an average size of 100 nm in diameter: this is consistent with the characteristic size range of exosomes (Figure 1A). By flow cytometry, we confirmed that the isolated nanostructures stained positive for canonical exosome markers CD9, CD63 and CD81 (Figure 1B).

### Profiling of exosomal and cellular miRNAs before and after Cetuximab treatment

**Figure 2:**
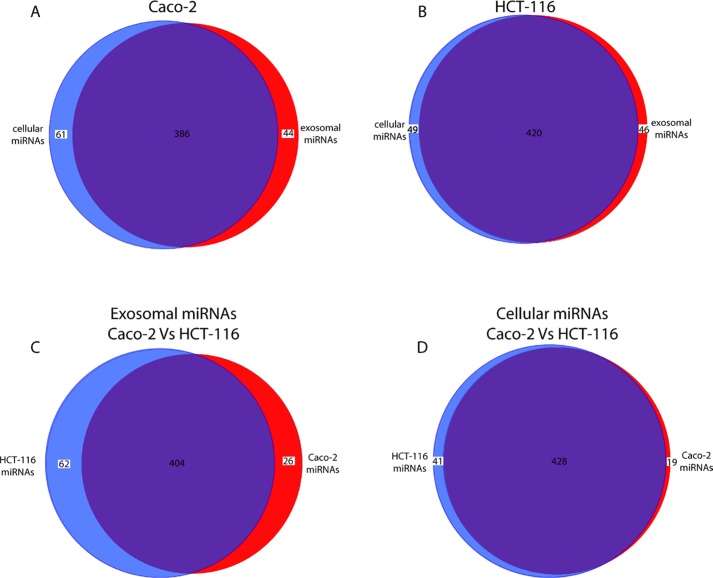
Comparison of miRNA sets found in exosomes and their source cells Venn diagrams show the overlap between miRNA sets in exosomes and their matching source cells: (A) Caco-2; (B) HCT-116. A third diagram (C) shows a comparison of miRNAs content in exosomes from Caco-2 and HCT-116 cells, respectively; (D) same data are shown for cellular miRNAs in Caco-2 and HCT-116 cells (for details see Materials and Methods).

**Figure 3:**
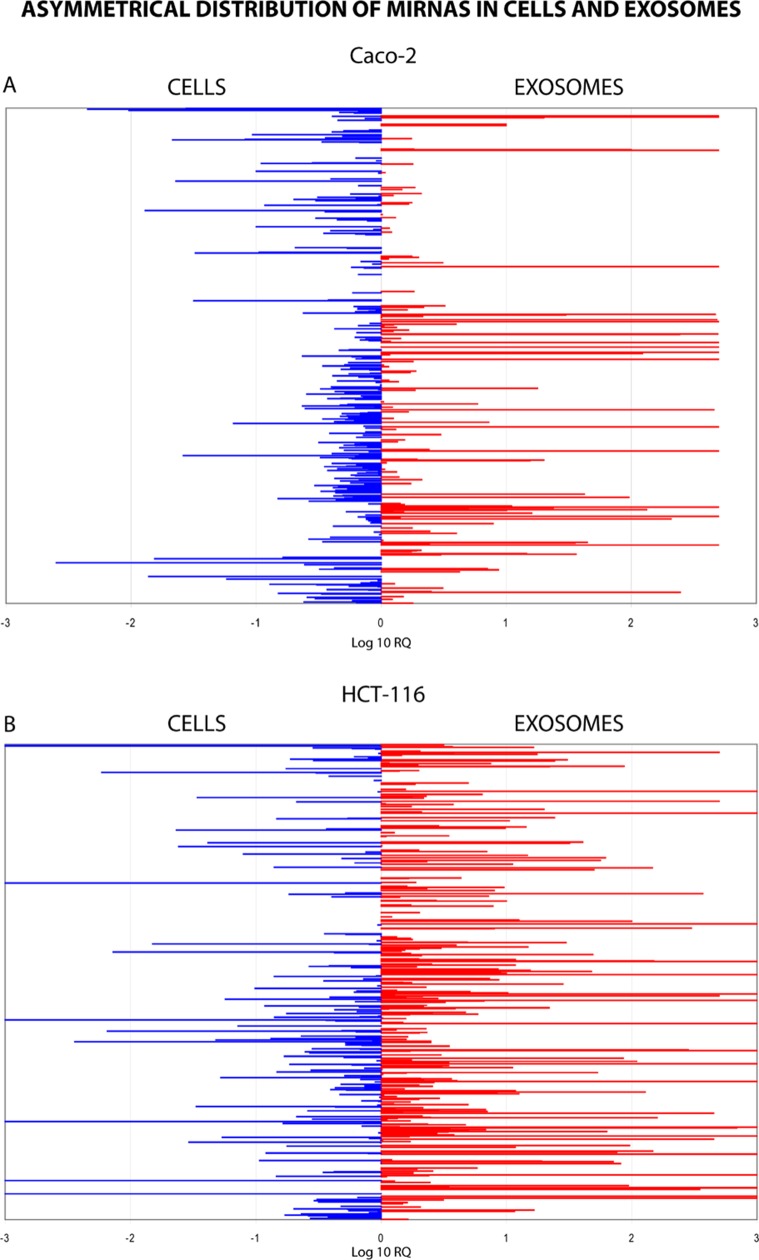
Quantitative asymmetric distribution of miRNAs in CRC cells and their exosomes Relative quantities (RQ) of exosomal miRNAs were compared to those from source cells: Caco-2 (A); HCT-116 (B). Values are shown as log10 of RQ.

Using TaqMan Low Density Array (TLDA) technology, we determined the expression profiles of 754 miRNAs in exosomes from Caco-2 and HCT-116 cells; through the analysis of the same samples, we also characterized the miRNA content in the respective source cells. In all cases, analysis was performed before and after seven days of Cetuximab treatment. We compared the sets of steady-state miRNAs in Caco2 cells (447 molecules detected), Caco2 exosomes (430), HCT-116 cells (469), and HCT-116 exosomes (466) (Figure 2). Both cell lines reciprocally shared about 93% of cellular miRNA species and about 90% exosomal miRNA species (Figure 2, C and D). Opposed to the overlap between exosomal and cellular miRNAs in the qualitative analysis, we detected a strong asymmetric distribution of miRNAs between secreted exosomes and their source cells when we subjected our data to quantitative analysis (Figure 3, A and B). Intriguingly, some miRNAs were found to be specifically located in exosomes (*eg*, miR-127, miR-136*, miR-144, miR-432, miR-433, miR-487b and miR-495 in Caco-2 exosomes; miR-136*, miR-223*, miR-380-5p, miR-432 and miR-672 in HCT-116 exosomes) (Figure 2, A and B). Steady-state exosomes from both cell lines were highly enriched in miRNAs involved in blocking proliferation and immune escape (*eg*, miR-142-5p, miR-150, miR-223, miR-433) (Table 1). Profiling of exosomal miRNAs after Cetuximab treatment showed changes for 25 and 20 miRNAs in Caco2 exosomes and source cells, respectively (Table 2 and 3); in comparison, we detected 9 and 12 miRNAs whose levels had been altered by Cetuximab in HCT-116 exosomes and source cells, respectively (Table 4). Notably, the steady-state asymmetric distribution of miRNA content in exosomes and their source cells was highly accentuated by Cetuximab treatment. Indeed, the sets of DE miRNAs in exosomes and source cells lines did not overlap qualitatively for both cell lines, with the exception of miR-31* that was moderately upregulated both in treated Caco-2 cells and their exosomes. Specifically, many DE miRNAs in the exosomes secreted by Caco2 also are involved in proinflammatory mechanisms in addition to performing cancer-related functions (*eg*, let-7a, miR-122, miR-133b, miR-511) (Table 2). In Cetuximab-treated Caco2 cells, we also detected upregulation of the CRC tumour-suppressors miR-1, miR-133a, miR-145 and miR-215 (Table 3). Considering both the number of DE miRNAs and the magnitude of changes, exosomal miRNA alterations in KRAS-mutated cells (HCT-116) were less pronounced (Table 4). Noteworthy was the downregulation of miR-624 and miR-1289, which are orphans of functions in CRC biopathology. Highly downregulated was also miR-802, a miRNA reported to perform a tumour suppressor role in intestinal epithelial cells (Table 4). The set of DE cytoplasmatic miRNAs in HCT-116 cells was characterized by moderate upregulation of several putative oncomirs (*eg*, miR-193a-3p, miR-424*, miR-501-3p, miR-938, miR-1285) (Table 4).

**Table 1:** Fold Changes and literature data of shared Caco-2 and HCT-116 DE miRNAs in exosomes versus cells at steady state FC: average Fold Change. PMID: PubMed ID. RQ value less than 1 were converted with the formula -1/RQ.

Shared Caco-2 and HCT-116 DE miRNAs in exosomes versus cellular pellets at steady-state				
miRNA	Caco-2 FC	HCT-116 FC	BioPathological Role	PMID
hsa-let-7e	−3.9	−4.1	It is downregulated in many cancers; it is involved in Taxol resistance.	19779035 23079871
hsa-miR-29a	−4.1	2.5	MiR-29a is overexpressed in CRC tissue and serum. MiR-29 suppresses immune responses to intracellular pathogens by targeting IFN-γ. It could be involved in tumor immune escape.	23673725 22426940 21785411 19584290
hsa-miR-100	247.2	−4.1	It can control proliferation and migration of endothelial cells by repressing mTOR pathway. It is downregulated in CRC tissues.	21339483
hsa-miR-136*	728.7	540.0		
hsa-miR-142-5p	132.8	8.0	MiR-142-5p is strongly expressed in T cells; it could have an immunosuppressive effect on B e T cells.	21343377 22043014 22549634
hsa-miR-144*	23.8	17.1	MiR-144* is overexpressed in CRC feces and could act as a immunosuppressor miRNA by negatively modulating T and B cells proliferation.	21863218 17604727 21367459
hsa-miR-150	96.0	205.1	MiR-150 is downregulated in CRC. It is upregulated in activated B e T cells, so it could be involved in immune cells normal development.	22052060 21590770 PMC3794731
hsa-miR-204	646.9	1.9	MiR-204, downregulated in CRC, can suppress head and neck tumor metastases. It is significantly upregulated in T cells activated by anti-CD3 antibodies in vitro.	20056717 20056717 22343615
hsa-miR-223	444.9	330.1	MiR-223 exogenous expression sensitizes breast and colon cancer cells expressing mutant p53 to treatment with DNA-damaging drugs and it could inhibit cell proliferation. Overexpression of miR-223 is related to granulocyte-specific lineage.	22043014 /23584479 22569260 18291670
hsa-miR-376c	907.7	8.6	It is downregulated in melanoma; it can promote tumorigenesis and metastasis. Potential serum biomarker for early detection of gastric cancer.	22747855 23922722 22432036
hsa-miR-411	223.8	9.2		
hsa-miR-432	243.8	829.6	Increased in plerixafor-mobilized CD34+ cells.	20818715
hsa-miR-433	492.5	287.8	MiR-433 increased inhibition of cell proliferation in HeLa cells treated with 5-FU. It is downregulated in gastric carcinoma. It can act to escape immune elimination.	23915286 19531230 20694010
hsa-miR-487b	484.2	15.2	Expression of miR-487b was found to be specific in sera of patients with CRC.	PMC2802653
hsa-miR-548J3	−30.6	−31.0	MiR-548 could be involved in immune suppression through IFN-γ1 downregulation during viral infections.	23150165
hsa-miR-744	−3.5	2.8	It is upregulated in CRC cancer stem cell. Down-regulated in sw620 respect to sw480. Potential serum biomarker for early detection of gastric cancer.	23552465 21934092 22169097
hsa-miR-1201	−72.3	−259.7		
hsa-miR-1274A	35.6	6.4		
hsa-miR-1274B	44.6	3.6		

**Table 2:** Fold Changes and literature data of exosomal DE miRNAs in Caco-2 after Cetuximab treatment

Exosomal DE miRNAs	Caco-2 FC	Literature data on BioPathological implications	PMID
hsa-let-7a	3.8	Tumor suppressor in CRC / Proinflammatory role.	16651716 20630862
hsa-miR-22*	1.6		
hsa-miR-29a	−1.5	MiR-29a is overexpressed in CRC tissues and serum. It suppresses immune responses to intracellular pathogens by targeting IFN-γ. It could be involved in tumor immune escape.	23673725 22426940 21785411 19584290
hsa-miR-31*	1.6	Downregulation or absence of miR-31 has been detected in human breast cancers. It regulates metastasis by opposing local invasion and metastatic colonization.	22429812 19524507
hsa-miR-34c	−11.7	MiR-34c was shown to be downregulated through promoter hypermethylation in colon cancer; the loss of expression was also consistent with data from colon cancer cell lines. Loss of miR-miR-34c helps metastatic cells to escape tumor immune surveillance.	22992310 23922103 22962263
hsa-miR-122	−2.7	Tumour suppressor gene in hepatocarcinogenesis: it affects Wnt/b-catenin-TCF signalling pathway; it also performs an anti-inflammatory role in liver. Reduction of miR-122 expression in macrophages is involved in immune escape.	22276989 22820288 24098824
hsa-miR-130a	−1.9	Oncomir whose overexpression enhances cell proliferation and migration.	23393589
hsa-miR-133b	46.2	MiR-133b regulates tumor cell proliferation and apoptosis through modulation of the MET signaling pathway in CRC. It could be involved in proinflammatory cytokine IL-17A expression in lymphocytes.	20505319 21637854
hsa-miR-151-5P	1.7	Upregulated in prostate cancer. Amplified in CRC and kidney cancer.	22928040
hsa-miR-182	−1.9	Mir-182 promotes proliferation and survival of CRC cells. miR-182-5p is induced by IL-2 and promotes T cell-mediated immune responses.	23019418 20935646
hsa-miR-184	−2.0	Overexpression of the oncomiR miR-184 might play an oncogenic role in the antiapoptotic and proliferative processes of tongue squamous cell carcinoma. Plasma miR-184 levels were associated with primary tumors.	18451220
hsa-miR-193a-3p	−2.2	Its expression was high in Malignant Pleural Mesothelioma compared to both renal cell carcinomas (RCC) and non-RCC carcinomas.	20864637
hsa-miR-194*	1.7		
hsa-miR-212	−1.9	Downregulated in human CRC tissues; it might prevent tumor progression.	23583431
hsa-miR-296	−1.7	Decrease in blood of miR-296 predicts chemotherapy resistance and poor clinical outcome in patients receiving systemic chemotherapy for metastatic coloncancer.	22892985
hsa-miR-409-5p	5.7	Tumor suppressor in gastric cancer.	22179828
hsa-miR-501-3p	−2.5	Differentially expressed in NFPA (non-functioning pituitary adenomas) compared to normal pituitary. Predicted to target immune suppressive genes	21063788
hsa-miR-505	−3.1	Tumor suppressor miRNA, which induces apoptosis in MCF7-ADR cells (a drug-resistant breast cancer cell line) in presence of docetaxel.	22051041
hsa-miR-511	18.6	Overexpression of miR-511-3p in BM-derived cells inhibits tumor growth; it is downregulated in CRC; it also is a putative positive regulator of Toll-like receptor 4 and initiator of innate immune response.	22832163 23459799 23437218
hsa-miR-518d	5.9	Potentially involved in cisplatin resistance of germ cell tumor cell lines.	21575166
hsa-miR-615-5p	5.3	Preferentially expressed in HCC, but not in normal livers. Its forced expression in HCC cell lines led to significant decrease in cell growth and migration through targeting IGF-II.	22819824
hsa-miR-885-5p	2.3	Upregulated in serum of patients with liver inflammatory pathologies.	20815808
hsa-miR-886-5p	4.5	Pre-miR-886 plays a putative tumor-suppressive role. It is upregulated in human NK cell activation through IL-2, IL-15 and IL-21 stimulations.	21518807 22701882
hsa-miR-1233	−2.1	Overexpressed in RCC patients; RCC-associated oncomir.	21984948
hsa-miR-1303	2.4	Upregulated in CRC cell lines after treatment with celecoxib.	22970014

**Table 3:** Fold Changes and literature data on cellular DE miRNAs in Caco-2 after Cetuximab treatment

Cellular DE miRNAs	Caco-2 FC	Literature data on BioPathological implications	PMID
hsa-miR-1	9.2	MiR-1 can have a tumor suppressor function in colorectal cancer by directly downregulating MET, impairing cell proliferation and motility.	22343615
hsa-miR-30b	1.9	Putative oncogenic target in medulloblastoma. miR-30b/30d regulation of GalNAc transferases enhances invasion and immunosuppression during metastasis.	21741600 19584924
hsa-miR-30d	1.6	Mir-30d regulates tumor cell proliferation, apoptosis, senescence, and migration. miR-30b/30d regulation of GalNAc transferases enhances invasion and immunosuppression during metastasis.	21741600 22058146
hsa-miR-31*	1.8	Downregulation or delection of the miR-31 genomic locus is found in human breast cancers. It regulates metastasis by opposing local invasion and metastatic colonization.	22429812 19524507
hsa-miR-33a	1.9	Involved in chemoradiotherapy response in individual tumor samples with rectal cancer.	18695884
hsa-miR-132	−2.0	Downregulated in CRC. miR-132 regulates antiviral innate immunity through suppression of the p300 transcriptional coactivator.	PMC3511678 20418869
hsa-miR-133a	2.9	MiR-133 inhibits cell proliferation, migration and invasion in prostate cancer cells by targeting EGFR.	22407299
hsa-miR-145	3.5	MiR-145 suppressescellinvasion and metastasisby directly targeting mucin 1 in breast and colon cancer cell lines.	19996288
hsa-miR-193b*	2.7		
hsa-miR-215	2.8	MiR-215 is highly expressed in colon cancer stem cells with slow proliferation rate and resistance to chemotherapy.	PMC2881118
hsa-miR-339-5p	2.3	MiR-339-5p is a tumor suppressor by regulating expression of PRL-1. It is downregulated in colorectal cancer tissues and highly invasive CRC cell lines.	23696794
hsa-miR-504	3.7	Mir-504 can directly regulate the tumor suppressor gene p53.	20542001
hsa-miR-564	−2.7		
hsa-miR-622	−2.8	Downregulation of miR- 622 in gastric cancer promotes cellular invasion and tumor metastasis by targeting ING1.	21528065
hsa-miR-663B	−2.9	Involved in CRC.	PMC1450142
hsa-miR-766	1.8	Upregulated in cutaneous squamous cell carcinoma patients biopsies.	23026055
hsa-miR-875-5p	−3.0		
hsa-miR-499	12.4		
hsa-miR-1271	3.4	Upregulated in head and neck cancer tissue.	21637912
hsa-miR-1276	−2.4		

**Table 4:** Fold Changes and literature data of exosomal and cellular DE miRNAs in HCT-116 after Cetuximab treatment

Exosomal DE miRNAs	HCT-116 FC	Literature data on BioPathological implications	PMID
hsa-miR-135b*	2.1	It belongs to a family of oncogenes involved in colorectal adenomas and carcinomas	22660396 18632633
hsa-miR-193a-5p	1.9	Overexpressed in medulloblastomas (associated with WNT signaling). Increased levels within exosomes released by the human mast cell line HMC-1	21358093 24009880
hsa-miR-194-5p	−1.5	MiR-194 is downregulated in colorectal cancer.	19074875
hsa-miR-296-5p	1.8	Upregulated in colorectal cancer.	16609010 24084739
hsa-miR-328	1.9	Downregulated in Colorectal Cancer.	PMC1550420 22453125
hsa-miR-624-3p	−39.2	Upregulated in fibroblasts in proliferative arrest and in conditions of quiescence / senescence with function of potential tumor-suppressor	19475566
hsa-miR-671-3p	2.3	Downregulated in colon cancer. Identified in exosomes of Epithelial cells and Lung cancer cells	23255074 20615901
hsa-miR-802	−22.0	Involved in adenocarcinoma. Overexpressed in colon and intestine.	20558762
hsa-miR-1289	−15.9		
Cellular DE miRNAs	HCT-116 FC	Literature data on BioPathological implications	PMID
hsa-miR-139-5p	1.6	Overexpressed in aggressive mucinous phenotype of CRC.	21739196
hsa-miR-193a-3p	3.1	Possible involvement in the development and progression of SCC (Squamous Cell Lung Carcinoma Tissues). miR-193a is strongly upregulated in CD4+ lymphocytes of relapsing-remitting multiple sclerosis patients.	20620595 20148420
hsa-miR-212-3p	−1.5	Downregulation of miR-212 is a possible mechanism of Cetuximab resistance in head and neck squamous cell carcinoma.	20856931
hsa-miR-424*	3.0		
hsa-miR-501-3p	4.7	Differentially expressed in NFPA (Non-Functioning Pituitary Adenoma) compared to normal pituitary. Predicted to target immune suppressive genes	21063788
hsa-miR-502-3p	1.6	Contributes to the early development of breast cancer. Upregulated in colorectal cancer stromal tissue.	19789321 22452939
hsa-miR-545*	6.4		
hsa-miR-548d-3p	−1.7	Upregulated in periodontitis tissues	22128589
hsa-miR-604	−60.8		
hsa-miR-652-3p	−1.7	Downregulated in squamous cell lung carcinoma tissues (SCC).	20508945
hsa-miR-938	2.2	Overexpressed in sporadic non-functioning pituitary adenomas.	21063788
hsa-miR-1285-3p	16.3	Downregulated in renal cell carcinoma	22294552

### Gene Ontology analysis of exosomal and cellular miRNAs in CRC cells before and after Cetuximab treatment

**Figure 4:**
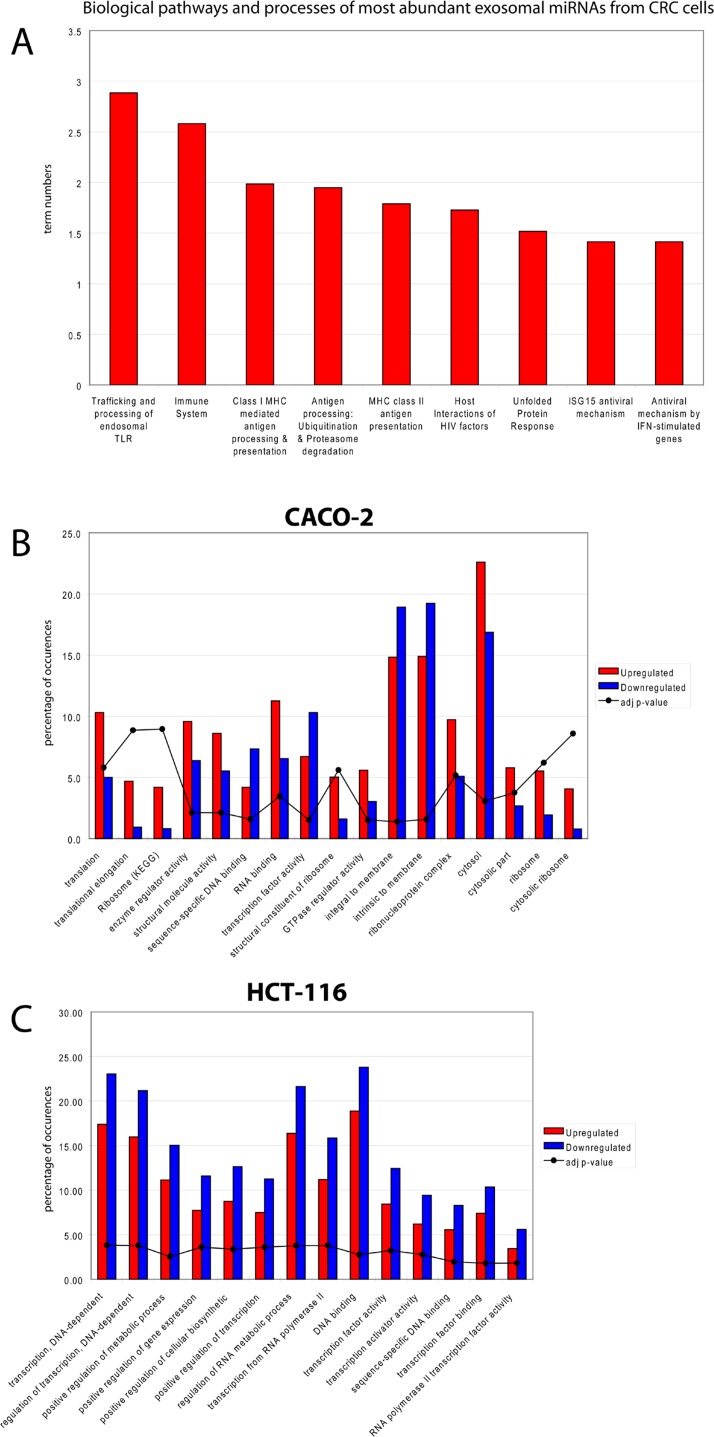
Biological functions of exosomal miRNAs in CRC (A) Prediction of the biological functions of the most abundant exosomal miRNAs. Based on their validated or putative targets, the related function was predicted for both cell lines at steady-state; GO annotations are shown that exhibited a significant overrepresentation in upregulated miRNAs with respect to those downregulated in exosomes from (B) Caco-2 and (C) HCT-116 cells after Cetuximab treatment.

**Figure 5:**
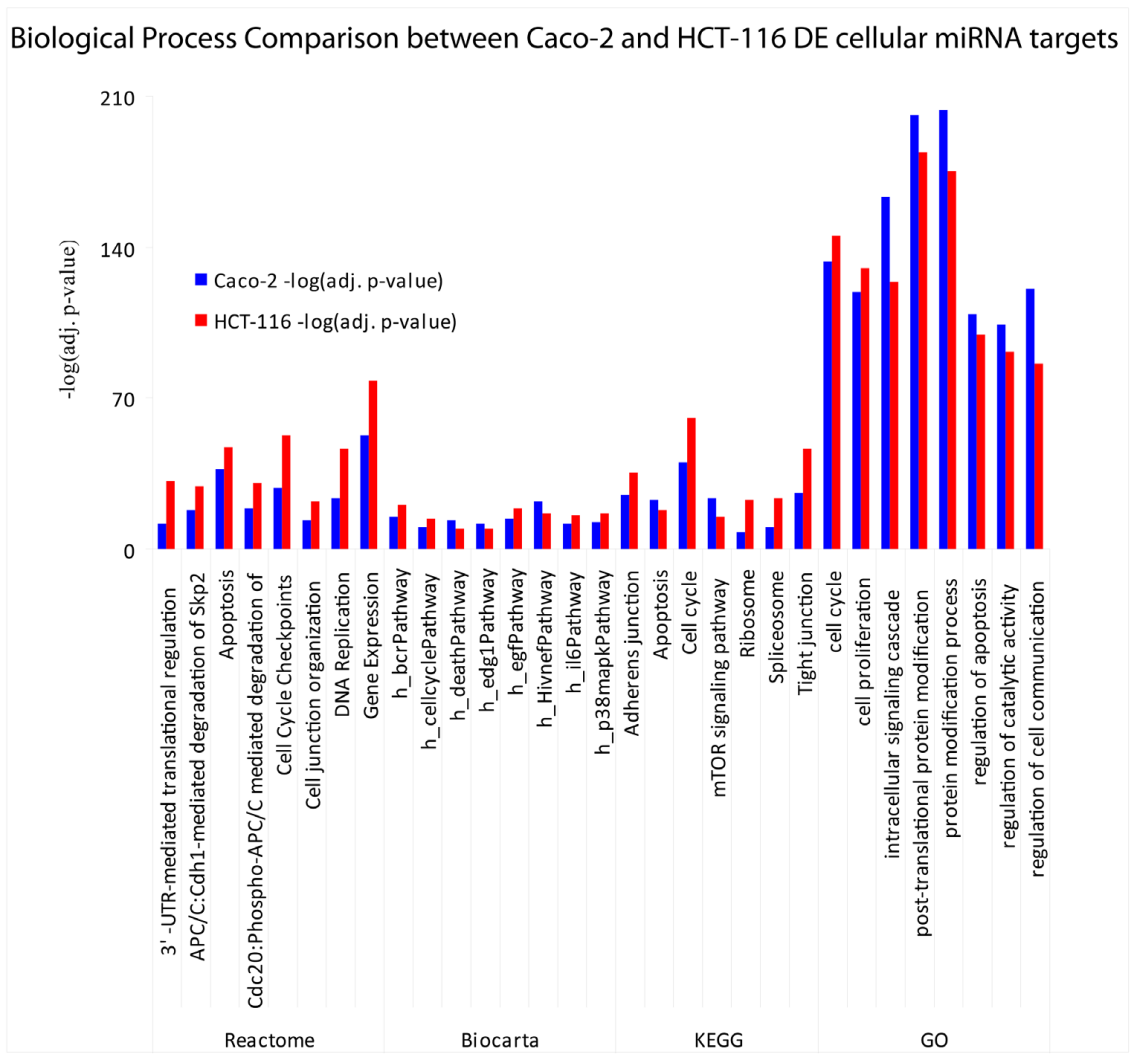
Comparison of biological functions attributed to cellular DE miRNAs in Caco-2 and HCT-116 cells after Cetuximab treatment A comparison is shown of biological terms from Biocarta, Gene Ontology, KEGG and Reactome that are associated with DE miRNAs in Caco-2 and HCT-116 cells after Cetuximab treatment. Values plotted in the histogram are show as –log10 of the adjusted p-value.

To infer the biological processes regulated by exosomal and cellular DE miRNAs at steady-state and after Cetuximab treatment, we retrieved validated and predicted miRNA targets; then, we statistically analysed their Gene Ontologies and pathways involvement by David and FatiGo tools. According to Reactome Database, targets of exosomal miRNAs from both cell lines at steady-state were statistically enriched in genes and proteins involved in modulation of the immune system (trafficking and processing of endosomal Toll-like receptors, antigen processing and presentation, unfolded protein response) (Figure 4A). This agrees with literature reports that the most abundant miRNAs in steady-state CRC exosomes are mainly involved in immune escape (Table 1). For treated Caco-2 cells, exosomal DE miRNAs were functionally enriched in biological processes as immunity, EGFR pathway, apoptosis, cell cycle (Table 5). Upregulated miRNAs were statistically more present in molecular processes associated with negative control of translation than those downregulated (Figure 4B). For treated HCT-116, exosomal DE miRNAs were enriched in biological processes related to apoptosis, cell cycle and immunity (Table 6). On the other hand, downregulated miRNAs showed a significant overabundance of GO terms related to transcription, positive regulation of transcription and positive regulation of metabolic processes when compared to overrepresented miRNAs (Figure 4C). Direct comparison of GO categories and pathways of the targets of DE miRNAs after Cetuximab treatment of Caco-2 and HCT-116 cells, showed several statistically significant differences: targets of DE miRNAs from Caco-2 cells were more represented in intracellular signalling and cell communication than those from HCT-116 cells; in HCT-116 cells, we observed a statistically significant expression of cell cycle and gene expression processes when compared to Caco-2 cells (Figure 5).

**Table 5:** Caco-2 DE exosomal miRNAs target pathway involvement Statistically pathways retrieved for each database are reported with the specific adjusted p-value between brackets.

Caco-2 pathway involvement	Biocarta (adj. pvalue)	Reactome (adj. pvalue)	Kegg database (adj.pvalue)	GO biological process (adj. pvalue)
Notch signaling pathway	h_notchpathway (1.49977E-3)	Signaling by Notch (2.49195E-10)	Notch signaling pathway (2.13939E-9)	Notch signaling pathway (2.96332E-8)
Immunity	h_tcrPathway (7.74677E-3)	Signaling in Immune system (1.24332E-2)	T cell receptor signaling pathway (2.6041E-7)	
	h_bcrPathway (2.1619E-2)		B cell receptor signaling pathway (2.6041E-7)	
	h_il2rbPathway (2.899E-2)			
Axon guidance		Axon guidance (2.02318E-7)	Axon guidance (3.26891E-8)	
EGFR signaling pathway	h_fMLPpathway (1.7231E-2)	Signaling by EGFR (9.59843E-4)	ErbB signaling pathway (1.291E-7)	regulation of cell proliferation (6.82672E-10)
	h_erkPathway (2.04915E-2)		MAPK signaling pathway (4.56095E-7)	positive regulation of cell proliferation (1.06127E-8)
	h_erbB4pathway (2.1619E-2)			regulation of developmental process (9.49053E-14)
	h_dspPathway (2.93048E-2)			cell morphogenesis involved in differentiation (2.44283E-7)
	h_cblPathway (3.68769E-2)			regulation of cell proliferation (6.82672E-10)
Signal trasduction	h_gpcrPathway (2.04915E-2)			regulation of signal transduction (2.20524E-7)
				positive regulation of small GTPase-mediated signal transduction (5.38885E-8)
Apoptosis	h_deathPathway (2.04915E-2)	Apoptosis (1.03569E-2)		negative regulation of apoptosis (6.82672E-10)
	h_fasPathway (0.0221267E-2)			negative regulation of programmed cell death (7.43189E-10)
	h_mef2dPathway (0.0368769E-2)			anti-apoptosis (5.58559E-7)
				regulation of programmed cell death (6.12346E-7)
Cell cycle	h_cdc42racPathway (2.49196E-2)	Cell Cycle, Mitotic (1.28553E-2)	Cell cycle (9.16981E-6)	cell cycle (1.63257E-7)
	h_rbPathway (3.68769E-2)			
	h_cdc25Pathway (2.49196E-2)			
Nerve growth factor (NGF)		Signalling by NGF (2.02318E-7)	Neurotrophin signaling pathway (3.10315E-8)	
Insulin signaling pathway		Signaling by Insulin receptor (3.99996E-3)	Insulin signaling pathway (1.89788E-4)	
Cell junction		Cell junction organization (8.31255E-3)	Tight junction (2.92519E-5)	
			Focal adhesion (9.02754E-5)	

**Table 6:** HCT-116 DE exosomal miRNA target pathway involvement Statistically significant pathways retrieved for each database are reported with the specific adjusted p-value between brackets.

HCT-116 pathway involvement	Biocarta (adj.pvalue)	Reactome (adj.pvalue)	Kegg database (adj. pvalue)	GO biological process (adj. pvalue)
Cell cycle	h_cellcyclePathway (4.87207E-5)		Cell cycle (1.22799E-2)	Interphase (1.59847E-3)
	h_p53Pathway (1.66826E-2)			
	h_p27Pathway (2.89184E-2)			
	h_srcRPTPPathway (2.89184E-2)			
Apoptosis	h_p53Pathway (1.66826E-2)	Apoptosis (2.96866E-2)		
Reproductive system development	h_carm-erPathway (1.19906E-2)			Urogenital system development (4.06633E-4)
Immunity	h_lymphocytePathway (2.03082E-2)		Hematopoietic cell lineage (2.48383E-2)	B cell homeostasis (1.87229E-3)
	h_monocytePathway (2.89184E-2)			
	h_vipPathway (2.89184E-2)			

### Profiling of exosomal proteins before and after Cetuximab treatment

Using antibody microarrays, we profiled the expression of 741 cancer-related exosomal proteins in Caco-2 and HCT-116, at steady-state and after Cetuximab treatment. For this analysis, 18 biological replicates were selected on the basis of their concentration, 5 controls and 4 treated samples each for both cell lines.

**Figure 6:**
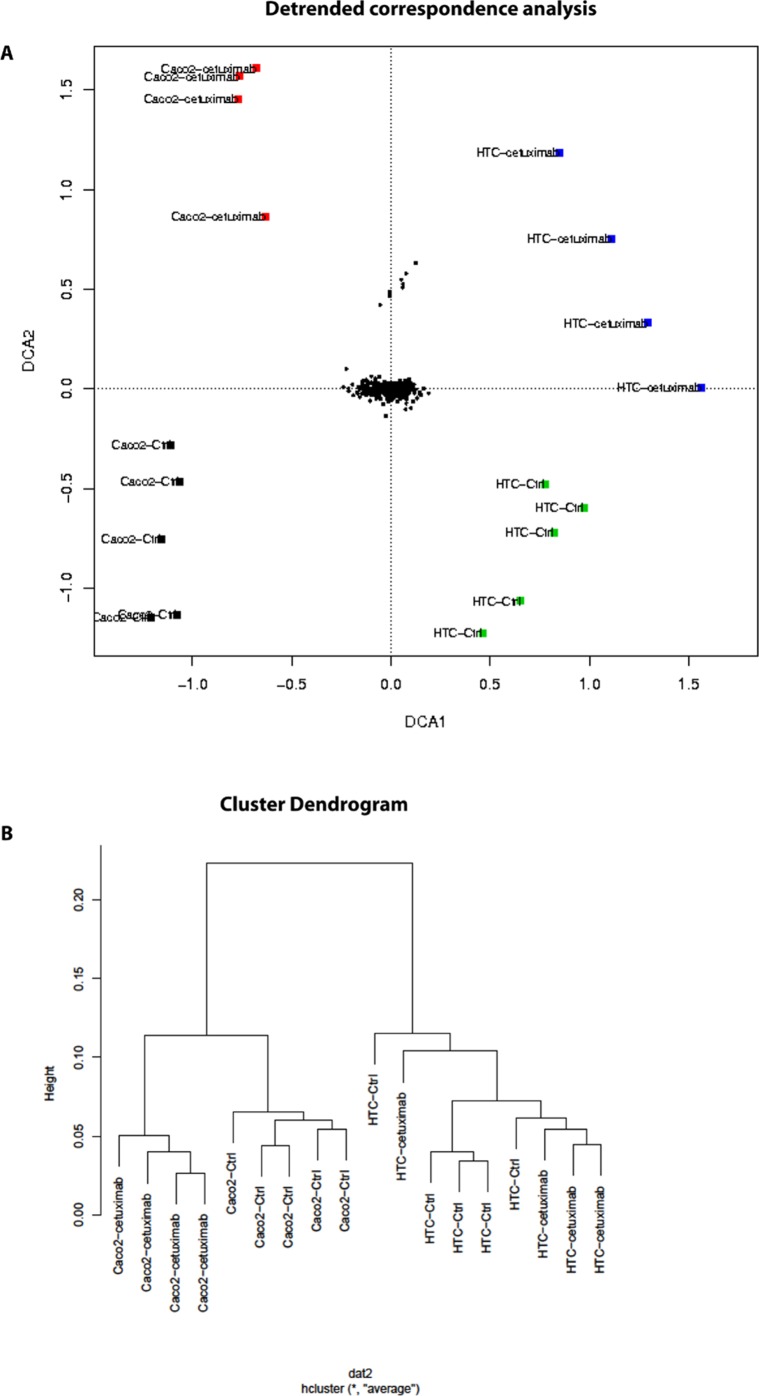
Antibody array data from CRC exosomes after Cetuximab treatment (A) Detrended correspondence analysis of array data from Caco-2 and HCT-116 exosomes before (Ctrl) and after treatment (Cetuximab). (B) Hierarchical clustering of the exosome samples.

Similarities and differences among the various sample groups were globally assessed using Hierarchical Clustering, Non-Metric Multidimensional Scaling, and Detrended Correspondence Analysis (Figure 6A). For both cell lines, treated samples and controls showed a unique and reproducible protein expression pattern. Normalized data are presented in a cluster dendrogram tree (Figure 6B). Statistically significant DE exosomal proteins for both cell lines are shown in Tables 7, 8 and 9. We found 54 exosomal DE proteins in Caco-2 compared to controls (27 overexpressed, 27 downregulated), and 9 exosomal DE proteins in HCT-116 (5 overexpressed and 4 downregulated). These data indicate different qualitative alterations of exosomal proteomes of the two cell lines, which exhibit different sensitivity to Cetuximab. Only VCAM1 (an important protooncoprotein involved in angiogenesis) was overexpressed in exosomes from both cell lines following treatment. The strongest overexpressed proteins in treated Caco-2 exosomes (*eg*, CD59, CRP, CTSD, HMMR, TRIM22) are involved as potential oncogenes in the pathogenesis of different cancers, including CRC (Table 8). In addition, some important protooncogenes involved in cell cycle control (*eg*, BRAF, CDC2, ERBB2) were moderately overexpressed. Among the most downregulated proteins, we found several confirmed or candidate tumour suppressor genes (*eg*, ID1, FAS, FPR1, IL10, PCGF2, VDR). Notably, some exosomal DE proteins are components either of intracellular macromolecular complexes or of organelles (*eg*, ribosomes: RPL7, RPL10A, RPS15; mitochondria: MRPL3, ATP5H). Most overexpressed proteins are involved in immune response, as T-cells activation and functions (CD59, IL12A) or the response to bacterial and viral infections (CTSD, TRIM22). Opposed to this, some downregulated proteins are involved in immune suppression through induction of apoptosis and antiinflammatory activity (*eg*, EP300, FAS, IL10). Important to stress, only about 50% of Caco-2 DE proteins were already annotated in Exocarta (http://www.exocarta.org/), a comprehensive database of human exosomal proteins; for all other proteins, this is the first report on their presence in exosomes, in particular for CRC cells. The low number of DE exosomal proteins in treated HCT-116 suggests that Cetuximab altered their exosomal proteome to a lower extent than in Caco-2 (Table 9); this could be a hint to the molecular basis of HCT-116 resistance to treatment. Among downregulated proteins, PTEN is a well known tumour suppressor in CRC, while CDC20, TNF and VCAM1 are important protooncogenes. Four DE proteins are involved in immunity and intercellular signalling (IL1B, MS4A2, PTEN, TNF) (Table 9).

**Table 7:** Exosomal Caco-2 downregulated proteins after Cetuximab treatment Log of Fold Changes, adjusted p-values, literature data, PubMed ID (PMID), and Exocarta annotations (Ex.An.) are reported.

Gene symbol	adj.pvalue	Log-FC	BioPathological Role	PMID	Ex.An.
TF	0.013383	−0.946	Decreased levels during inflammation	10633294	Yes
TSPAN16	0.019818	−0.904			No
IL10	0.044541	−0.8015	The immunosuppressive cytokine IL-10 is associated with poor prognosis in colon cancer. More advanced stages of CRC are characterized by low IL-12p40 and high IL-10 serum levels. IL-10 is an anti-inflammatory cytokine, involved in infections immune response.	21972680 15034082 18424693 22428854	Yes
FPR1	0.029566	−0.79	FPR1 is involved in inflammation. It could be indirectly involved in the activation or suppression of immune response.	21216225 20539176	No
ID1	0.029566	−0.7715	ID1 and ID3 can control colon cancer-initiating cells self-renewal through cell cycle inhibitor p21, preventing the accumulation of DNA damage. Silencing of ID1 and ID3 sentitizes the cells to oxaliplatin treatment. Involved in metastasis formation of gastric cancer. It can block B-cell development at the early pro-B cell stage.	22698403 16271072 21200383	No
KRT4	0.032729	−0.744			Yes
ALB	0.040051	−0.6565			Yes
VDR	0.048814	−0.5915	It could influence colorectal cancer risk. VDR controls the level of nuclear β-catenin in colon cancer cells. It could increase CRC cells drug response. Anti-inflammatory activity.	18086783 21858154 17721433 20639756 PMC3166406	No
PCGF2	0.023829	−0.584	It could act as a tumor suppressor in cancer; loss of PCGF2 could increase breast cancer stem cells tumorigenicity.	22954590 20170541	No
DHX40	0.029566	−0.551			No
LMAN2	0.04983	−0.5355			Yes
ID3	0.048791	−0.5025	ID1 and ID3 can control colon cancer-initiating cells self-renewal through cell cycle inhibitor p21, preventig the accumulation of DNA damage. The silencing of ID1 and ID3 sentitizes the cells to oxaliplatin treatment. Involved in metastasis formation of gastric cancer. It can block B-cell development at the early pro-B cell stage.	22698403 16271072 21200383 10454544	No
BRPF3	0.039927	−0.4775			Yes
NAT13	0.038472	−0.474			Yes
MRPL3	0.048056	−0.4685			No
FAS	0.032729	−0.4665	Fas-induced apoptosis could be involved in tumor progression and drug response. Fas loss-of-function commonly accompanies the malignant phenotype. Anti-inflammatory through proapoptotic activity.	12204527 11733771 19239902	Yes
PAK1	0.044541	−0.4215	Rac1/PAK1 cascade controls β-catenin activation in colon cancer cells. It promotes prostate tumor growth and microinvasion. Involved in the regulation of immune cells motility and migration.	21822311 23258534 PMC3137287	No
NCL	0.029566	−0.417	Overexpressed in breast cancer, it can control miRNAs involved in breast cancer initiation, progression, and drug resistance. Potential oncogene.	23610125	No
RPL10A	0.032729	−0.378			Yes
RPS15	0.032729	−0.353			No
ALDH9A1	0.048791	−0.3435			Yes
SPINT2	0.040622	−0.3435	Putative tumor suppressor gene in medulloblastoma and implicated in the dysregulation of the HGF/MET signaling pathway. Downregulated in CRC.	19047176 23110343	No
MOXD1	0.043556	−0.329			No
TNPO3	0.048056	−0.3195	It is required for HIV infection; probable anti-immune function	PMC3599327	Yes
SLC29A1	0.044541	−0.2965	Potentially involved in cancer chemotherapy.	Yes	
EP300	0.048814	−0.289	EP300 is mutated in epithelial cancers (comprised CRC); it could act as a tumor-suppressor gene. Potential immune-suppressor role.	10700188 / 23955711	No
ADAM9	0.048814	−0.278	Overexpression of ADAM9 promotes colon cancer cells invasion.	23514059	Yes

**Table 8:** Exosomal Caco-2 upregulated proteins after Cetuximab treatment Log of Fold Changes, adjusted p-values, literature data, PubMed ID (PMID), and Exocarta annotations (Ex.An.) are reported.

Gene symbol	adj.pvalue	Log-FC	BioPathological Role	PMID	Ex.An.
CD59	0.019818	1.597	It could be involved in anti-cancer immune response through T cells activation in CRC.	19380765	Yes
TRIM22	0.040622	1.5935	TRIM22 has been implicated in cellular differentiation and proliferation and may play a role in certain cancers and autoimmune diseases. It could play an antiproliferative role in cancer. Involved in anti-viral protection through INF.	22649727 19218198	No
CTSD	0.039927	1.3395	CTSD is essential for the dissemination of pancreatic cancer cells in vivo. Could be involved in anti-microbial response.	21948970 22337873	Yes
IGHA1	0.029566	1.2505	Immunoglobulin heavy constant alpha 1.		Yes
VCAM1	0.026082	1.202	Upregulation of VCAMI might prevent disruption of cell-cell interactions and, hence, colorectal cancer dissemination. Promotion of immune response and T-cell mediated inflammation.	9495363 18216105	No
CRP	0.048232	1.167	Involved in chronic low-grade inflammation that is correlated with increased risk of CRC.	16489056	No
RPL7	0.029566	1.129	Expressed in Thyroid Carcinoma.	21509594	No
HMMR	0.006069	1.081	It is widely upregulated in human cancers and correlates well with cell motility and invasion.	22203674	No
OVGP1	0.032729	1.045	Potential markers for ovarian epithelial cancers.	20130498	Yes
LY6K	0.024522	1.017	Upregulation in bladder cancer. LY6K is a cancer biomarker and a therapeutic target that induces invasion and metastasis.	PMC3031884 22988241	No
PIR	0.027041	1.0135			No
HLA-DMB	0.031823	0.9525	Expression of HLA-DMB is associated with improved survival in advanced-stage serous ovarian cancer. Expressed in APC cells and generally involved in the immune response.	PMC3000165 GENE	Yes
FUS	0.029566	0.935	Depletion of FUS reduced androgen-dependent proliferation of prostate cancer LNCaP cells	21909421	Yes
EZR	0.040051	0.922	Ezrin could be considered as a biomarker for the progression of cervical lesions. In pancreatic cancer cell lines, Ezrin silencing decreased cellular protrusions/microvilli formation, anchorage-independent growth, cell migration and invasion.	23067217 23324233	Yes
CDC2	0.032729	0.8055	Cdk2/cdc2 is remarkably upregulated together with a malignant change in CRC.	9664116	Yes
IL12A	0.040622	0.7785	This cytokine is required for T-cell-independent induction of interferon (IFN)-gamma, and is important for the differentiation of both Th1 and Th2 cells.		No
CD81	0.029566	0.7725	Exosomal marker for three CRC cell lines, potentially involved in hepatocellular carcinoma. Epigenetic inactivation of CD81 is a common feature of gastric tumors advantaging growth and survival of tumor cells.	22895844 11278880 23264205	Yes
BRAF	0.048814	0.71	Mutation in the BRAF oncogene is a key event in CRC pathogenesis.	22228154	Yes
ATP5G2	0.032729	0.5685			No
CLU	0.029566	0.561	It could represent a diagnostic molecular marker for colon cancer screening. Generally involved in innate immunity.	19879422 23493296	Yes
ATP5H	0.039792	0.5505			Yes
RCC1	0.040051	0.5285			No
ERBB2	0.029566	0.448	Potential oncogene. Activation of ERBB2 signaling causes resistance to the EGFR-directed therapeutic antibody Cetuximab.	21900593	Yes
AGR2	0.038472	0.4285	AGR2 is expressed in most human adenocarcinomas and could support tumor growth. It induces expression of amphiregulin. Increased AGR2 and LGR5 are associated with poor outcomes in CRC.	21454516 22605983	Yes
EWSR1	0.040051	0.364	Breakage or translocations of EWSR1 are involved in different tumors within control of cell growth, differentiation and proliferation.	PMC3586390	No
RIN1	0.027041	0.3425	Strongly expressed in LoVo colon cancer cell line.	22812185	No
MALL	0.038472	0.299			No

**Table 9:** Exosomal HCT-116 DE proteins after Cetuximab treatment Log of Fold Changes, adjusted p-values, literature data, PubMed ID (PMID), and Exocarta annotations (Ex.An.) are reported.

Gene symbol	adj.pvalue	Log- FC	Cancer and/or immunity involvement	PMID	Ex.An.
PTEN	0.021436	−1.541	The tumor suppressor PTEN expression level (down-regulated) may provide valuable prognostic information to aid treatment strategies for colorectal cancer patients. Involved in Cetuximab response.	19036165 18339877	No
MS4A2	0.009843	−1.209	Beta subunit of the high affinity IgE receptor. Involved in allergy.	Gene - NCBI	No
IL1B	0.023891	−1.0465	IL1B plays a critical role in the early onset of tumor-associated angiogenesis. Polymorphisms in IL1B as well as IL1B haplotype analysis may serve as molecular markers for tumor recurrence in stage II CRC. Involved in TNF-signaling promotion.	18987561 23487424	No
CACNA1G	0.046921	−1.044	In CRC, CACNA1G inactivation may play a role in cancer development by modulating calcium signaling, which potentially affects cell proliferation and apoptosis.	10493502	No
PRKCG	0.023891	1.082			No
VCAM1	0.026082	1.202	Upregulation of VCAMI might prevent disruption of cell-cell interactions and, hence, colorectal cancer dissemination. Promotion of immune response and T-cell mediated inflammation	9495363 18216105	No
TNF	0.026082	1.302	CRC-derived TNF-α can stimulate VEGF-A and MMP-2 production by macrophages to promote colon cancer cells angiogenesis. Involved in Cetuximab response of EGFR+ cells. Promotion of immune response.	17283136 23849826 17992258	No
CDC20	0.023891	1.365	Upregulated in CRC cell lines and primary cancer tissue. It predicts a poor diagnosis for CRC patients.	23758705	No
MAD2L1	0.046921	1.3685	Mutated and upregulated in breast cancer cell lines	11066082	No

### Network and canonical pathway analysis of exosomal proteins in CRC cells before and after Cetuximab treatment

**Figure 7:**
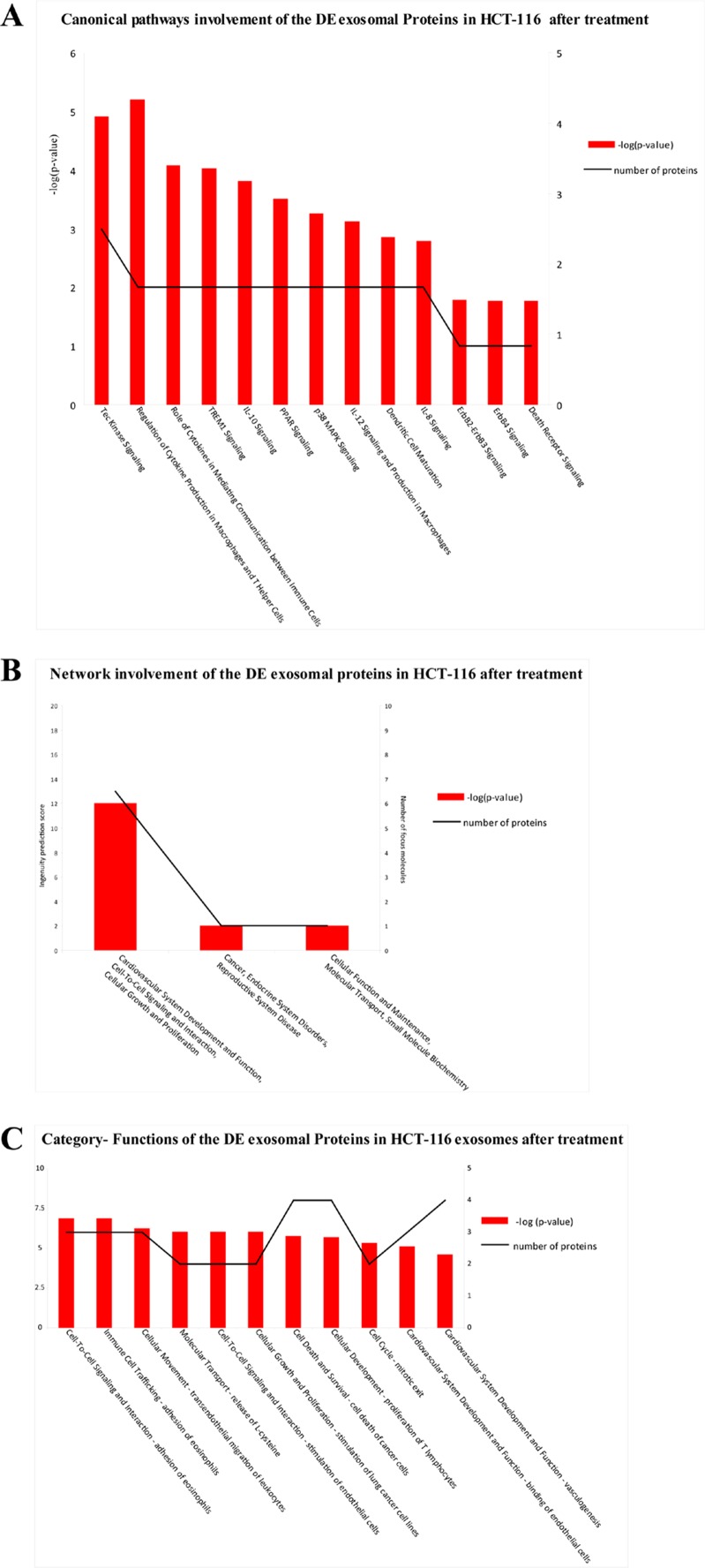
Biological functions of DE exosomal proteins in HCT-116 Analysis of biological functions associated with DE exosomal proteins from Cetuximab-treated HCT-116 cells based on an Ingenuity System analysis: (A) canonical pathways; (B) network involvement; (C) functional annotation categories.

**Figure 8:**
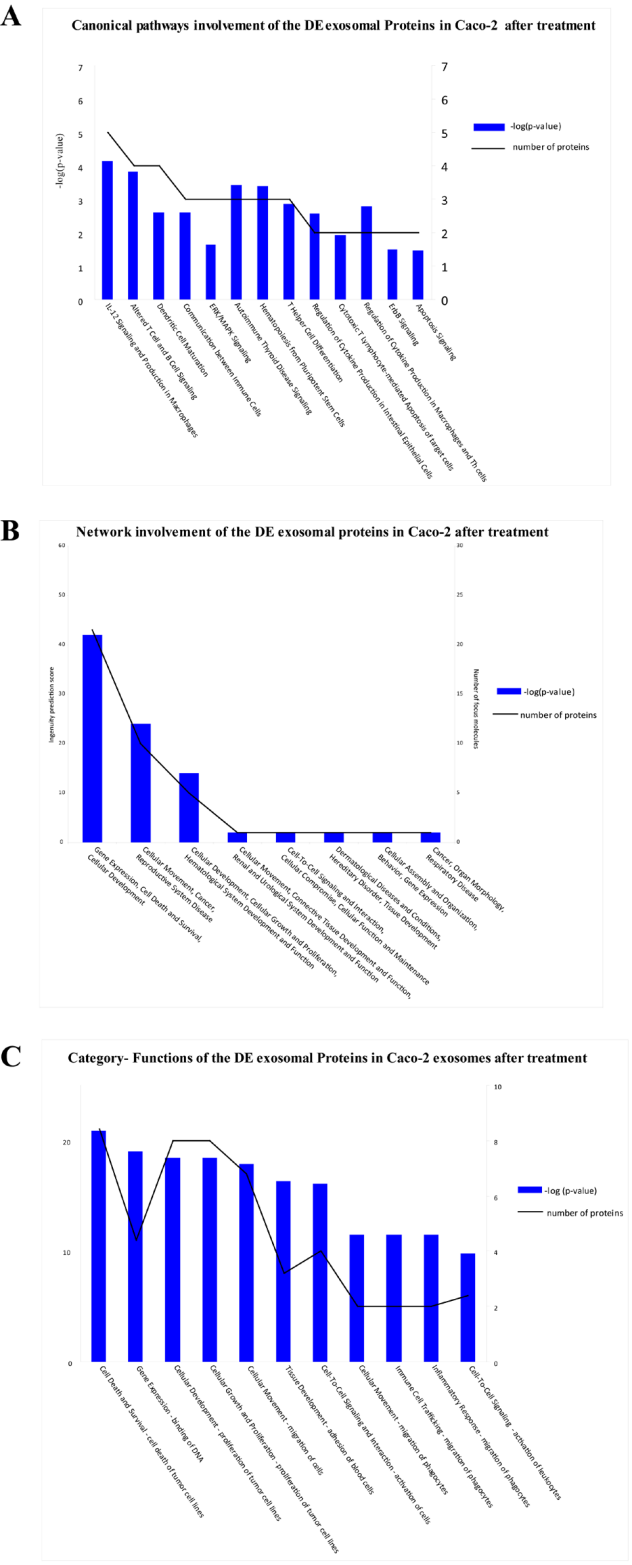
Biological functions of DE exosomal proteins in Caco-2 Analysis of biological functions associated with DE exosomal proteins from Cetuximab-treated Caco-2 cells based on an Ingenuity System analysis: (A) canonical pathways; (B) network involvement; (C) functional annotation categories.

DE exosomal proteins were analyzed to determine their GO classification and pathway involvement through Ingenuity software. Network and canonical pathway analysis for Caco-2 exosomal DE proteins yielded particularly relevant results, as they showed their important involvement in cell growth and proliferation, intercellular signalling, and cell death (Figure 8, A, B, C); also well represented are the pathways related to immune response (*ie*, IL-12 signalling and production in macrophages, T- and B-cell signalling in immune diseases, T helper cell differentiation and activation of leukocytes). The same analysis for HCT-116 exosomes demonstrated that most DE proteins are involved in biological processes as cell to cell signalling, cell growth and proliferation, cell death, and cell movement (Figure 7 A and B). A small number of DE proteins is involved in immune cell trafficking and inflammatory response (Figure 7C).

### Modifications of cell viability following exosomes transfection

**Figure 9:**
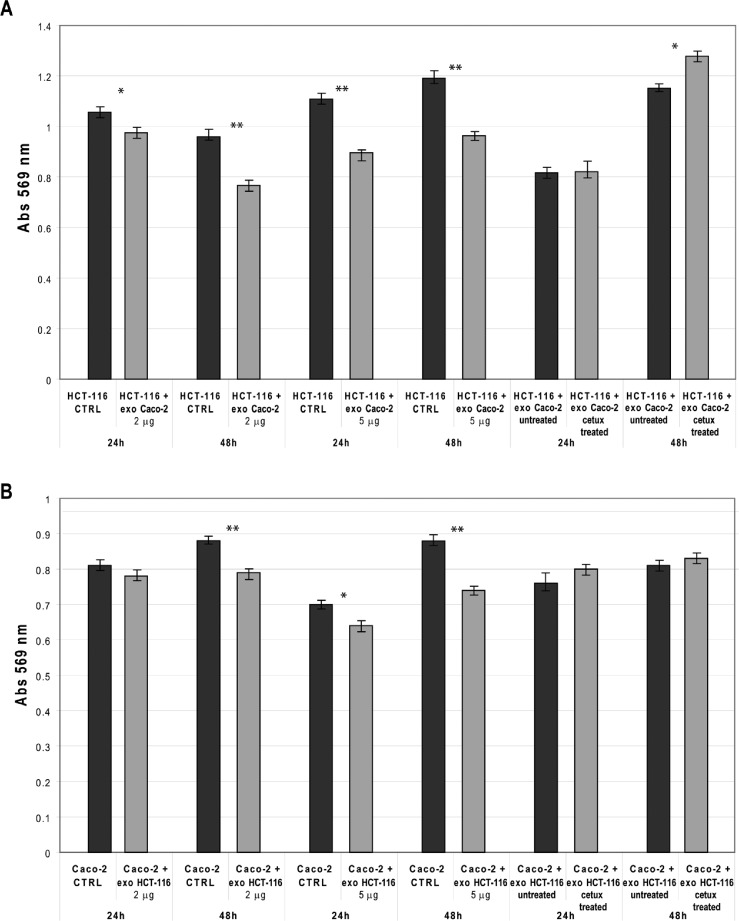
CRC cells viability after incubation with exosomes from untreated and Cetuximab-treated cells (A) MTT analysis of HCT-116 cells incubated for 24 h and 48 h with exosomes. Left and centre: untreated cells (CTRL) were compared to cells to which 2 μg or 5 μg, respectively, of exosomes from Caco-2 cells at steady-state had been added. Right: differences of incubations with exosomes from Caco-2 cells with or without Cetuximab treatment. (B) Results of an MTT assay on Caco-2 cells incubated for 24 h and 48 h. Left and centre: untreated cells (CTRL) were compared to cells to which 2 μg or 5 μg of exosomes from HCT-116 cells at steady-state had been added. Right: differences of incubations with exosomes from HCT-116 cells with or without Cetuximab treatment. CTRL: solvent used for exosome resuspension (PBS). Unpaired t-test, * P ≤ 0.05; ** P ≤ 0.01.

**Figure 10:**
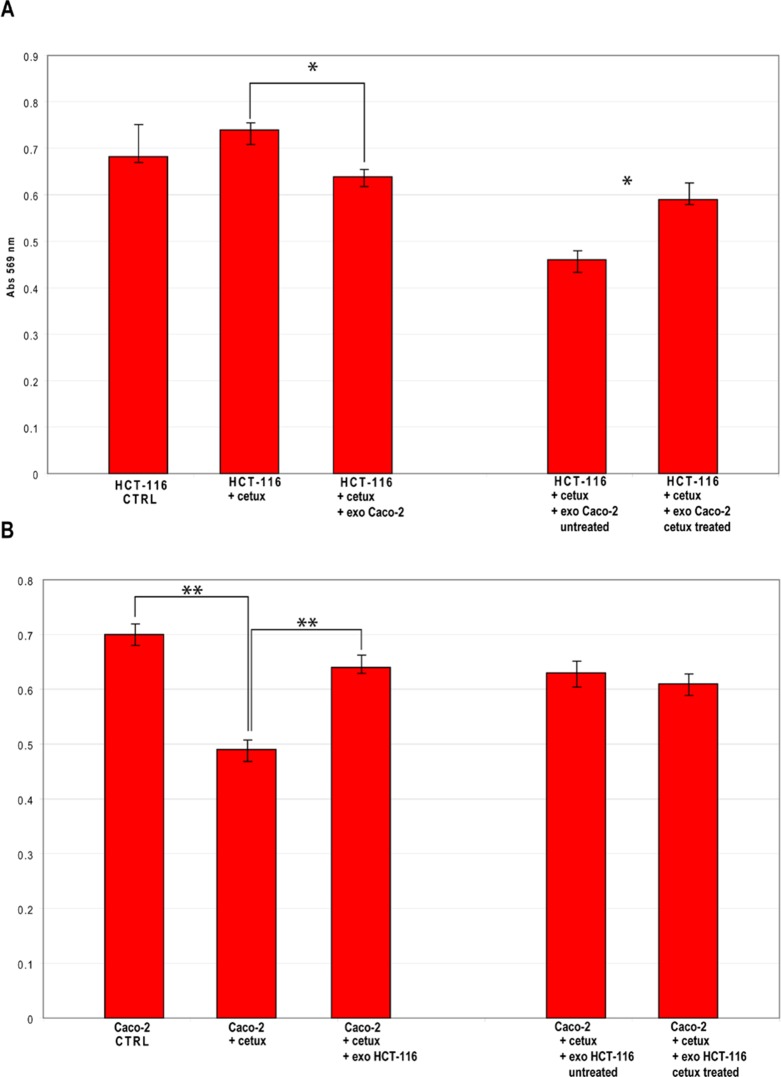
Viability of CRC cells simultaneously treated with Cetuximab and exosomes from untreated or treated cells (A) Results of MTT assays on HCT-116 cells treated with Cetuximab for 24 h and co-incubated with 5 μg of exosomes from Caco-2 cells that had been treated or not with Cetuximab. (B) Results of MTT assays on Caco-2 cells treated with Cetuximab for 24 h and co-incubated with 5 μg of exosomes from HCT-116 cells that had been treated or not with Cetuximab. CTRL: solvent used for Cetuximab resuspension (PBS). Unpaired t-test, * P ≤ 0.05; ** P ≤ 0.01.

To analyze the biomolecular effects of exosomes secreted by CRC cells before and after Cetuximab treatment, we transfected HCT-116 cells with exosomes from steady-state or Cetuximab-treated Caco-2 cells and checked HCT-116 viability at 24 h and 48 h after transfection (AT). In turn, Caco-2 cells were transfected with steady-state or Cetuximab-treated HCT-116 exosomes and analyzed the same way. HCT-116 treated with 2 μg of steady-state Caco-2 exosomes showed a slight decrease of viability at 24 h AT, which became more evident at 48 h AT. By using 5 μg of exosomes, the differences of viability were even more pronounced (Figure 9A). Also Caco-2 cells that had been incubated with HCT-116 exosomes exhibited significantly lower proliferation rates at 48 h AT (Figure 9B). When HCT-116 cells were transfected with exosomes from treated Caco-2, we observed no significant variation at 24 h AT; proliferation rates increased significantly at 48 h AT in comparison to HCT-116 cells transfected with exosomes from untreated Caco-2 (Figure 9A). Caco-2 cells transfected with exosomes from treated HCT-116 showed no significant difference in cell viability compared to controls (Figure 9B). Treatment with Cetuximab of HCT-116 cells, which had been transfected with 5 μg of steady-state exosomes, decreased their viability (Figure 10A). In contrast, Caco-2 cells simultaneously incubated with Cetuximab and exosomes from steady-state HCT-116 increased their viability compared to Caco-2 cells, which had been treated only with Cetuximab (Figure 10B). Notably, cell proliferation increased when we administered Cetuximab to HCT-116 cells incubated with exosomes from Cetuximab-treated Caco-2 cells (Figure 10A). We found no appreciable difference of viability when Caco-2 cells were treated with Cetuximab and transfected with exosomes purified from treated HCT-116 cells (Figure 10B). Overall, these data suggest that exosomes from untreated cells decreased cell viability, when incubated with recipient cells different from their source cells. In constrast, exosomes from Cetuximab-treated cells induced a proliferation increase only when purified from Caco-2 cells, but not from HCT-116. It is worth to stress that simultaneous incubation with Cetuximab and exosomes altered viability of both cell lines (Figure 10A and B).

## DISCUSSION

### Molecular cargo of exosomes from Caco2 and HCT-116 at steady-state

When they were first discovered, exosomes were considered *garbage bags* used by cells to get rid of unneeded or dangerous molecules. However, following the characterization in the mid-1990s of extracellular vesicles from antigen-presenting lymphocytes, exosomes were associated with immune system functions [34 – 36]. In recent years, many reports have convincingly demonstrated an important function of exosomes: they work as shuttles transporting signalling molecules (*eg*, mRNAs, miRNAs, proteins) involved in intercellular communication [15]. Intercellular exchange of nucleic acids and proteins via exosomes has been shown to be an effective mechanism of intercellular communication, especially within the tumor microenvironment [37]. Indeed, transport of RNAs and proteins from tumor cells to neighbouring cells and *vice versa* could have significant effects on their molecular phenotype. For instance, in tumorigenesis it could modulate proliferation, invasion and cell immunoreactivity. An important protumorigenic role could be performed by tumor-derived exosomes through their involvement in drug resistance: (1) exosome secretion could be utilized by cancer cells to expel anticancer drugs; (2) surface molecules from cancer-derived exosomes could compete for binding with antibody-based drugs, so lowering their therapeutic efficacy [33, 38]. Quite surprisingly, no data have been published to date on an important biological and translational issue: could the molecular composition of exosomal cargo be modulated by drug treatment? In this work, we have demonstrated significant alterations of both exosomal miRNAs and oncoproteins cargo in CRC cells following treatment with anti-EGFR antibodies. These molecular alterations did not precisely mirror the changes that occurred at the same time in source cells. Specifically, we analyzed exosomal miRNAs and proteins from Cetuximab-treated Caco-2 (sensitive) and HCT-116 (resistant) cells. Indeed, more than 90% of miRNAs expressed at steady-state conditions were shared by exosomes and their source cells for both cell lines. However, a sizeable fraction of miRNAs was exosome-specific. Most important, the asymmetric quantitative distribution of miRNAs between exosomes and cells, already present in both cell lines at steady-state, was significantly increased by treatment with Cetuximab. Interestingly, exosomes from both lines shared miRNAs whose amount was highly altered in comparison to source cells. Several upregulated exosomal miRNAs had been previously reported to be differentially expressed in CRC (*eg*, miR-144*, miR-150, miR-204, miR-411, miR-487b); some of them have potential immunosuppressive activity on T- and B-cells (*eg*, miR-142-5p, miR-144*, miR-150, miR-433). Extracellular miR-144* was found to be overexpressed in feces of CRC patients, suggesting that it could be a candidate diagnostic marker for non-invasive tumour detection [39]; this miRNA also could be involved in regulating antituberculosis immunity through modification of cytokine synthesis and reduced proliferation of T-cells [40]. Expression levels of miR-150 decreased during progression of CRC tumors [41]; its enforced expression in colon HT29 cells lowered c-Myb and Bcl-2 levels, thus enhancing cell apoptosis [42]. MiR-150 is upregulated in mature resting B- and T-cells, while it is strongly downregulated in their cell precursors and upon immune activation [43]; its overexpression in hematopoietic stem and progenitor cells impairs B-cell and T-cell development [44]. MiR-433 inhibits HeLa cell proliferation following treatment with 5-FU; it is downregulated in gastric carcinoma [45, 46]; it also reduces NK cell cytotoxicity and acts synergistically with other miRNAs to allow immune control escape [47]. Gastric cancer patients with high frequency of recurrence and poor survival outlook showed low levels of miR-142-5p [48]; in contrast, its expression is elevated in peripheral blood leukocytes from IL-10(−/−) mice during minimal colon inflammation [49]; inhibiting miR-142-5p in healthy donor CD4+ T-cells caused T-cell overactivation and B-cell hyperstimulation, whereas its overexpression in Systemic Lupus Erythematosus CD4+ T cells had the opposite effect [50]. Taken together, these data suggest that most upregulated miRNAs in exosomes from Caco-2 and HCT-116 cells show a dual biological function: (i) negative regulation of proliferation or apoptosis induction in cancer cells; (ii) immunosuppression in B- and T- cells. This agrees with our biologic data showing a decreased proliferation of Caco-2 cells after transfection with HCT-116 derived exosomes and *vice versa*. This anticancer effect of tumor-derived exosomes on tumor cells was already reported for other tumors, but not for CRC: exosomes secreted by human pancreatic tumor cells increased Bax and decreased Bcl-2 expression, inducing the mitochondrial apoptotic pathway in pancreatic cancer cells [51]. Moreover, these exosomes also decreased expression of the intranuclear target of Notch-1 signalling pathway and thereby negatively modulated its prosurvival functions [51]. This somehow unexpected proapoptotic role of cancer-derived exosomes could be explained by hypothesizing a negative autoregolatory loop of their own growth: tumors could exploit this strategy to favor the recruitment of endothelial cells and the establishment of neovasculature. Assuming that the functions of CRC exosomes, which we observed *in vitro*, mimic those occurring *in vivo*, it may be hypothesized that apoptotic and antiproliferative activities of exosomes also are addressed to immune cells to escape the immune system, in addition to cancer cells to regulate the tumor growth rate [51]. Notably, our GO analysis on targets of exosomal miRNA from both cell lines at steady-state showed a statistical enrichment in proteins involved in the modulation of the immune system. Diverse immunosuppressive effects of tumor-derived exosomes have been reported. Tumor-derived exosomes (including those from CRC cells) were shown to directly suppress the activity of effector T-cells by activating the expression of death ligands (*eg*, FasL, TRAIL), which can trigger the apoptotic death of activated T-cells [52, 53]. Interestingly, when CRC cells were treated with Cetuximab and incubated with exosomes from untreated cells, we observed an alteration of their viability. Specifically, cell vitality decreased slightly in Cetuximab-treated HCT- 116 cells incubated with Caco-2 exosomes; in contrast, we observed an increase of viability in Cetuximab-treated Caco-2 cells incubated with HCT-116 exosomes. This latter phenomenon is biologically consistent with results reported by Ciravolo et al. and Battke et al. on Trastuzumab-treated breast carcinoma cells [33, 54]: exosomes secreted by HER2-overexpressing breast carcinoma cell lines express HER2 molecule, enabling them to bind to HER2 antibody Trastuzumab both *in vitro* and *in vivo*; these exosomes efficiently bound and sequestered tumor-reactive antibodies and dramatically reduced their binding to tumor cells: in this way, they inhibited the overall effect of Trastuzumab on cancer cells proliferation. Given that exosomes express different forms of EGFR, the molecular target of Cetuximab [55 - 57], extracellular binding between Cetuximab and exosomal EGFR could reduce antibody binding to CRC cancer cells, partially neutralizing the effects of antibody-based therapeutics.

### Exosomes molecular cargo in Caco2 and HCT-116 cells after Cetuximab treatment

Exosomes represent a vesicle-based mechanism of cancer cells to signal changes to the microenvironment of a developing tumor. Accordingly, it is not surprising that pharmacologically-treated cancer cells alter the content of molecular signals carried inside the exosomes. Indeed, our data demonstrate that the asymmetric expression of miRNAs in exosomes and cytoplasm, already existing at steady-state, was significantly accentuated after Cetuximab treatment: exosomal miRNAs differentially expressed after treatment did not overlap with those detected in source cells. These data suggest that miRNAs alterations inside the exosomes were not due to an *osmotic effect* of expression variation of the cytoplasmic miRnome, but they were specifically induced in the nanovesicles. More specifically, we found 25 DE miRNAs in exosomes from treated Caco-2 cells involved as oncomirs or tumour suppressors in many cancer-related functions. Intriguingly, most dysregulated miRNAs (let-7a, miR-122, miR-133b, miR-511) had been already reported to participate in inflammatory processes. Let-7a negatively controls IL-13, a cytokine essential for allergic lung disease, an experimental model of asthma [58]. In liver, miR-122 deficiency causes inflammation and fibrosis that lead to hepatocarcinogenesis [59]. A tight coregulation with the miR-206/133b cluster induces proinflammatory cytokine IL-17A in lymphocytes; moreover, miR-133b was found in inflammatory microvesicles in association with metabolic and cardiovascular diseases [60, 61]. MiR-511 is a putative positive regulator of Toll-like receptor 4, an important initiator of native immune responses and inflammation [62]. Interestingly, our data on exosomal protein profiles from treated Caco-2 showed an enrichment of upregulated proteins involved in tumor etiopathogenesis, including CRC (CD59, CRP, CTSD, HMMR, LY6K, TRIM22), and in cell cycle control (BRAF, CDC2, ERBB2). However, some of these proteins also are involved in immune response and inflammation, as T-cells function and activation (CD59, IL12A) [63, 64]; others are involved in the response to bacterial and viral infections (CTSD, TRIM22) [65, 66]. On the other hand, we found FAS, FPR1, ID1, IL10, PCGF2, VDR among downregulated proteins: all are involved in tumor immune-escape through induction of apoptosis and anti-inflammatory activities [67]. Not surprisingly, analysis of networks and canonical pathways of exosomal DE proteins in treated Caco-2 cells showed again a general association of these proteins with the immune response (*eg*, IL-12 signalling and production in macrophages, T- and B-cell signalling in immune diseases, T helper cell differentiation, activation of leukocytes). Taken together, these data suggest that the molecular exosomal cargo (miRNAs and proteins), secreted by Caco-2 after treatment with Cetuximab, mainly carry proinflammatory signals. This observation on exosomes from Cetuximab-sensitive cells is potentially important, considering that response to this drug is often associated with immune activation and inflammation in CRC patients. In previous work, it was reported that most of CRC cellular DE miRNAs at 24 and 48 h after treatment controlled targets related to immune activation [32, 68]. Moreover, several studies have shown that in tumor microenvironment inflammation contributes to proliferation and survival of malignant cells, angiogenesis, metastasis, subversion of adaptive immunity, reduced response to hormones and chemotherapeutic agents [69 - 71]. Accordingly, exosomes could contribute with their miRNAs and proteins to induce *in vivo* proinflammatory phenomena in CRC treated patients; these would result in a protumoral behaviour. This latter hypothesis is supported by our results on significantly increased cell viability of HCT-116 (at steady-state and after Cetuximab treatment) after incubation with exosomes from treated Caco-2 cells. As already reported in the Results section, miRNAs and proteins alterations in exosomes from Cetuximab-treated HCT-116 were less striking than those detected in Caco-2, both for the number of DE molecules as for the magnitude of the changes. This difference could be due to the Cetuximab resistance of these cells. Indeed, we observed no relevant variation of cell viability when Caco-2 cells were incubated with exosomes from treated HCT-116 cells.

## CONCLUSIONS: BIOMOLECULAR, GENETIC AND TRANSLATIONAL IMPLICATIONS

During tumor progression, cancer cells modulate their molecular phenotype through genetic and epigenetic mechanisms [72, 73]. These alterations also involve qualitative and quantitative changes of the exosomal cargo, which in turn affect the microenvironment (this paper and cited bibliography). The predominant biomolecular functions of exosomes depend on their specific molecular phenotype as on that of their source cells. In addition, environmental factors seem to have an important role in determining the behaviour and immunologic impact of tumor-derived exosomes. When pharmacologically treated, CRC cells specifically alter miRNAs and proteins exosomal profiles. The overall biological effects of these alterations are related to *in vitro* drug resistance and tumor survival, but they also are likely to reflect *in vivo* phenomena occurring in patients. Careful *in vivo* molecular analysis of serum exosomes from large-scale epidemiological studies on cohorts of CRC patients before and after Cetuximab treatment is warranted before clinical applications. Intriguingly, knowledge of how exosomes could influence the tumour environment *in vivo* during therapy could pave the way to develop procedures of exosome block or removal as an additional therapeutic strategy in CRC patients [74]. Switching to a wider scientific horizon, our results and those from the literature also confirm that important genetic and biologic features could be horizontally transferred among eukaryotic cells through exosomes and their molecular cargo: these potentially paradigm-changing perspectives should be carefully investigated [75, 76].

## MATERIALS AND METHODS

### Cell lines

Caco-2 and HCT-116 cells are from the Interlab Cell Line Collection (ICLC), an international Repository Authority within the IRCCS Azienda Ospedaliera Universitaria San Martino-IST *Istituto Nazionale per la Ricerca sul Cancro* (Genova, Italy, EU). Caco-2 cells were cultured in Eagle minimal essential medium (EMEM) (Cambrex Bio Science), supplemented with 20% fetal bovine serum (FBS), 2 mM L-glutamine, 1% NEAA (Gibco); HCT-116 cells were cultured in McCoy 5A medium (Gibco), supplemented with 10% FBS and 2 mM L-glutamine (Gibco). Characterization and validation of cell lines were performed by the cell repository: cell lines were verified to be mycoplasma-free by Hoechst staining and PCR (TIB Molbiol), and by MycoTect (Gibco BRL); species verification was performed by isoenzyme analysis (AuthentiKit TM System, Innovative Chemistry): multiplex short-tandem-repeat profiling confirmed identity and uniqueness of cell lines. After receiving cells from ICLC, an aliquot was cultured up to the 10^th^ passage to perform the experiments; remaining cells were immediately frozen at −196°C. FBS was depleted of exosomes by centrifugation for 70’ at 120,000 g followed by filtration through 0.2 μm filters.

### Cetuximab treatment

A total of 5 × 10^6^ or 6 × 10^6^ cells were seeded in 175 cm^2^ culture flasks in serum starvation conditions (1% FBS) for CaCo-2 and HCT-116, respectively. Cells were cultured for seven days and treated with 40 μg/mL Cetuximab (Erbitux^®^, Merck KGaA, Darmstadt, Germany) each second day. On the seventh day of treatment (PT), exosomes were isolated from culture media (see below). RNAs and proteins were extracted from the same aliquots of exosome preparations as from the same aliquots of source cells, for both treated and untreated samples (the latter were treated with an equivalent volume of PBS, the solvent of Cetuximab). All experiments were performed in biological triplicates.

### Exosome isolation, characterization and transfection

Exosomes were extracted from cell culture supernatants by centrifugation at 300 *g* to pellet debris, and then at 16,500 *g* for 30’, followed by filtration through a 0.2 μm filter. The final supernatant was ultracentrifuged at 120,000 *g* on a Beckman L8-70M ultracentrifuge in a SW28 rotor for 70’. Exosome pellets were resuspended in 300 μl PBS for FACS analysis or directly lysed for RNA and protein isolation.Exosomes from Caco-2 and HCT-116 CRC cells were analysed by: (1) Zetasizer Nano ZS (Malvern Instruments, UK); (2) flow cytometry for size determination and surface markers characterization, as previously reported [77]. Aldehyde/sulfate latex beads (Invitrogen, Sweden) (140,000) were incubated with 200 μl Caco-2 and HCT-116 exosomes at 3700B0;C for 30’ and then at 400B0;C for 16 h on a rotator apparatus. After centrifugation at 4,000 *g* for 10’, pellets were resuspended in 100 μl PBS; 20 μl of 1 M glycine were added to block unspecific binding sites at 20°C for 30’. After one wash with PBS and 1% FBS, exosome-coated beads were incubated with PE-conjugated CD9, CD63 or CD81 antibodies or isotype controls (BD Biosciences) for 60’ at 400B0;C. For FACS analysis, samples were washed and resuspended in 200 μl PBS/FBS and analysed with FACSCantoII (Becton Dickinson, San Diego CA) and FlowJo software (TreeStar). For cell viability assays, exosomes from treated or untreated source cells were isolated as described, resuspended in PBS and quantified by Qubit. 2 μg/100 μl or 5 μg/100 μl of exosomes were added to 9 × 10^3^ recipient cells in 96 well plates and incubated for 24 h and 48 h. Cell viability was assessed through an MTT assay, 24 h and 48 h following exosome transfection. Absorbance values were read with a *Multiscan Ascent* microplate reader (Thermo Fisher Scientific). All MTT experiments were performed in six biological replicates. The statistical significance was evaluated by t-test (p-value < 0.05).

### RNA isolation, reverse transcription and miRNA profiling by TaqMan Low Density Array

Total RNA was extracted with TriZol (Invitrogen), according to manufacturer instructions, and quantified by Qubit (Invitrogen). Samples (100 ng and 25 ng of total RNA from whole cell pellets or their exosomes, respectively) were retrotranscribed and preamplified [78]. Amplified products were loaded onto microfluidic cards of the TaqManHuman MicroRNA Array v3.0 A and B (Applied Biosystems). Experiments were performed in biological triplicates with a 7900HT Fast Real Time PCR System (Applied Biosystem). Result validation was obtained by single TaqMan assays (Applied Biosystems), according to the manufacturer's instructions.

### Analysis of miRNA expression data

To accurately profile miRNAs, we identified the most appropriate genes within the arrays that could be used for a reliable normalization of CRC cells or their exosomes. By applying two different methods, DataAssist v.3 software (Applied Biosystems) and the geNorm Algorithm [79], we pinpointed the following endogenous controls: miR-106a, miR-135b, miR-532-3p (Caco2 cells, panel A); miR-183*, miR-200a*, miR-1274B (Caco2 cells, panel B); miR-16, miR-138, miR-193b, (Caco2 exosomes, panel A); miR-9*, miR-183*, miR-766 (Caco2 exosomes, panel B); miR-185, miR-345, miR-362 (HCT-116 cells, panel A); miR-34a*, miR-1180, miR-1254 (HCT-116 cells, panel B); miR-106b, miR-135b, miR-301b (HCT-116 exosomes, panel A); miR-30a-3p, miR-148b*, miR-1274A (HCT-116 exosomes, panel B). MiRNAs expression changes were calculated by applying the 2^−ΔΔCT^ method and using the above-mentioned miRNAs as endogenous controls. Differentially expressed (DE) miRNAs were identified by SAM (Significance of Microarrays Analysis) (http://www.tm4.org), applying a two-class paired test among ΔCt of treated and control samples (cells and exosomes) by using a p-value based on 100 permutations; imputation engine: K-nearest neighbours (10 neighbours); false discovery rate < 0.15. We accepted as reliable only DE miRNAs concordant by using all endogenous controls. Expression data in the Result section are shown as average relative quantity (RQ) of all values, calculated with each endogenous control respect to the calibrator sample. RQ values < 1 were converted to negative changes by following the formula: −1/RQ.

### MiRNA targets analysis

Targets of DE miRNAs were identified by using a combination of different approaches: (i) structural predictions (http://mirecords.biolead.org); (ii) expression anticorrelation between miRNAs and their validated or putative mRNAs targets (http://mirgator.kobic.re.kr); (iii) data from Argonaute cross-linked immunoprecipitation-sequencing (CLIP-Seq) (http://starbase.sysu.edu.cn). The biological networks of DE miRNAs targets were built by retrieving the corresponding interactome data through NCBI Entrez Utilities Web Service Client. We determined the Gene Ontology (GO) functional classification of miRNA target networks through the tools DAVID (http://david.abcc.ncifcrf.gov) and FatiGO (http://babelomics3.bioinfo.cipf.es).

### Antibody microarrays

To investigate the alterations of Caco-2 and HCT-116 exosomal protein profiles following Cetuximab treatment, proteins were extracted, labelled and incubated on antibody microarrays to detect abundance differences. This highly sensitive technology permits a proteomic analysis of small amounts of samples isolated from tissues, cultured cells or body liquids [80-82]. We used an array of 1,800 features, representing 810 antibodies, which are directed against 741 cancer-related proteins, and various controls [83] (Additional_file_1). Array production, quality control and handling were performed as described in much detail earlier [83, 84].

### Exosomal protein extraction

Exosomal proteins were isolated from 28 different biological replicates (7 treated samples and 7 controls for both cell lines). Proteins were isolated by using an optimized lysis buffer [84]: 20% glycerol, 0.05 M bicine pH 8.5, 0.15 M NaCl, 0.002 M EDTA^.^2Na, 20 mM phenylmethanesulfonyl fluoride, 2% NP-40S, 1% sodium cholate, 0.25% n-dodecyl-β-D-maltoside (GenaXXon Bioscience, Ulm, Germany), 0.5% amidosulfobetaine-14, 1.0 U/μl of Benzonase (Merck Biosciences, Schwalbach, Germany), Halt Protease and Phosphatase Inhibitor Cocktail (Thermo Scientific, Bonn, Germany). Samples were incubated with 25 μl of lysis buffer for 1 h at 4°C on an orbital shaker, then they were centrifuged at 15,000 rpm at 4°C for 15’. The supernatant was aspirated with a fine needle, carefully avoiding to disturb the upper layer or the pellet. Protein concentration in the supernatant was determined with the Pierce BCA Protein Assay Kit using a NanoDrop ND-1000 spectrophotometer (Thermo Scientific).

### Protein labelling

Extracted protein samples were selected on the basis of their concentration; we studied only samples with a concentration of at least 1 mg/ml. If the value was higher, the protein concentration was adjusted to 1 mg/ ml. Four biologic replicates of treated samples and five of controls were analyzed for both cell lines, making a total of 18 samples. For each sample, 20 μg protein were labelled as described in detail earlier [83, 84], by using the fluorescence dye DY-649 (Dyomics, Jena, Germany). A second fluorescence dye, DY-549, was used to label a pool of protein extracts from human serum samples of healthy people. The pool acts as a reference to normalize signal intensities in a dual-colour analysis mode [85]. Labelling reactions were performed at a molar ratio dye-protein of 7.5, with the assumption that 60 kDa is the average molecular weight of all proteins. Labelling was carried in the dark in 0.1 M carbonate buffer, pH 8.5, at 4°C for 2 h. Unreacted dye was quenched with 10% glycine for 20’ at 4°C on an orbital shaker in the dark. Labelled samples were stored at −20° C until analysis.

### Sample incubation

Incubation of the microarrays with labelled samples was performed as reported [84]. In short, the antibody arrays were soaked in TBST buffer. Subsequently, samples were washed once for 5’ with TBST containing 0.05% Tween-20 (TBST20) (pH 6.5), followed by another wash for 15’ with TBST20 (pH 7.5). Slides were blocked in 5.0 ml of 10% non-fat dry milk (Biorad, Munich, Germany), at 20°C for 3 h, using Quadriperm chambers (Greiner Bio-One, Frickenhausen, Germany) on an orbital shaker. Blocked slides were incubated in Quadriperm chambers with 20 μg of DY-649 labelled sample and 20 μg of DY-549 labelled pool reference in 4 ml incubation buffer, containing 1% milk in PBST20 (pH 7.5), on an orbital shaker in the dark at 4°C for 16 h. Slides were washed four times, 5’ each, in large volumes of PBST20 (pH 7.5), rinsed with deionised water for 10’, and dried in a ventilated oven at 29°C. Scanning of the arrays was performed with a ScanArray-4000XL (Perkin Elmer,Waltham, USA) at constant laser power and photomultiplier tube gain (PMT). Images were analysed with the software GenePix Pro 6.0 (Molecular Devices, Sunnyvale, USA).

### Antibody microarray data analysis

GPR files of scanned images were analysed with Chipster software (v1.4.6, CSC, Finland). Data were normalized using normexp with background correction offset [0,50], as reported [86]. Similarities and differences among different sample groups were globally assessed using hierarchical clustering, non-metric multidimensional scaling [87], and detrended correspondence analysis [88]. Two-group comparisons (*ie*, Caco-2 cells *versus* HCT-116 cells, both for treated and control exosome samples; treated samples *versus* controls from the same cell line) were performed using the Empirical Bayes test with Bonferroni–Hochberg multiple testing correction; cut-off was set at a p-value of 0.05 [89]. Multiple-group comparisons (*ie*, for cell of origin and degree of differentiation) were performed using LIMMA with a p-value adjustment according to Bonferroni–Hochberg multiple testing correction [89]. Cluster analysis was conducted using Pearson correlations; dendrograms were constructed using the average linkage method (http://chipster.csc.fi). Functional investigations were performed with the Ingenuity Systems Pathway Analysis tools (Ingenuity Systems, Redwood City, CA). The p-values were calculated using right-tailed Fisher's exact test (www.ingenuity.com).
